# Calibration and Validation of a Wrist- and Hip-Worn Actigraph Accelerometer in 4-Year-Old Children

**DOI:** 10.1371/journal.pone.0162436

**Published:** 2016-09-12

**Authors:** Elin Johansson, Lisa-Marie Larisch, Claude Marcus, Maria Hagströmer

**Affiliations:** 1 Department of Neurobiology, Care Sciences and Society, Karolinska Institutet, Stockholm, Sweden; 2 Department of Clinical Science, Technology and Intervention, Karolinska Institutet, Stockholm, Sweden; Leeds Beckett University, UNITED KINGDOM

## Abstract

**Introduction:**

To determine time spent at different physical activity intensities, accelerometers need calibration. The aim of this study was to develop and cross-validate intensity thresholds for the Actigraph GT3X+ accelerometer for wrist and hip placement in four-year-old children.

**Methods:**

In total 30 children (49 months, SD 3.7) were recruited from five preschools in Stockholm. Equipped with an accelerometer on the wrist and another on the hip, children performed three indoor activities and one free-play session while being video recorded. Subsequently, physical activity intensity levels were coded every 5^th^ second according to the Children’s Activity Rating Scale. Receiver Operating Characteristic (ROC) curves was used to develop wrist and hip intensity thresholds, the upper threshold for sedentary, and lower threshold for moderate-to-vigorous physical activity (MVPA), for the vertical axis (VA) and for the vector magnitude (VM). A leave-one-out method was used to cross-validate the thresholds.

**Results:**

Intensity thresholds for wrist placement were ≤ 178 (VA) and ≤ 328 (VM) for sedentary and ≥ 871 (VA) and ≥ 1393 (VM) counts/5 seconds for MVPA. The corresponding thresholds for hip placement were ≤ 43 (VA) and ≤ 105 (VM) for sedentary and ≥ 290 (VA) and ≥ 512 (VM) for MVPA. The quadratic weighted Kappa was 0.92 (95% CI 0.91–0.93) (VA) and 0.95 (95% CI 0.94–0.96) (VM) for the wrist-worn accelerometer and 0.76 (98% CI 0.74–0.77) and 0.86 (95% CI 0.85–0.87) for the hip-worn.

**Conclusion:**

Using wrist placement and the VM when measuring physical activity with accelerometry in 4-year-old children is recommended.

## Introduction

Physical activity is associated with a broad range of health indicators and is probably already of importance during the preschool years [1]. An accurate method to measure physical activity and sedentary time is essential to study correlates of these behaviors and to evaluate interventions, both in research and in clinical settings. Accelerometers have revolutionized the research field by enabling objective measures of physical activity intensity, frequency and duration, which are possible to use in large cohorts [2]. Objective methods are of particular importance for preschool children who can neither recall nor estimate past physical activities, and whose intermittent activity patterns make proxy reports very difficult [3]. Accelerometers have been proven feasible, reliable and valid for assessing physical activity in children [4, 5]. Accelerometers can be worn on different body sites. The hip has been the traditional location, but during recent years the wrist has been increasingly used as it has been shown to yield superior compliance in children, compared with hip placement [6]. Accelerometer output, expressed as counts, is affected by the body part it is attached to. As an example, an accelerometer placed on the hip does not capture arm movements. However, the estimated time in different intensities is similar when site-specific cut-points are applied [7]. To determine the time spent at different intensity levels, accelerometer counts need to be calibrated against a reference method. In children, observational methods such as The Children’s Activity Rating Scale (CARS) [8] have been suggested as the best reference method and CARS has been used in many previous calibration studies [5, 9, 10]. Taken the large difference in children’s motor skills between the age of one and six year there is a need for age-specific cut-points. Calibration studies for wrist-placement have been performed in two-year-olds [5] and in 8–12 year olds [11] but cut-points for children aged 3–5 are lacking.

The aim was to calibrate the accelerometer Actigraph GT3X+ for non-dominant wrist and hip placement in four-year-old children by developing upper vertical axis (VA), and vector magnitude (VM), intensity thresholds for sedentary behavior and lower VA and VM intensity thresholds for moderate-to-vigorous intensity physical activity (MVPA). Further aims were to cross-validate the developed intensity thresholds and to test the sensitivity and specificity of two existing cut points for hip-worn accelerometers.

## Method

### Subjects

A feasibility sample of preschools (n = 5) located in the inner-city and suburbs of the Stockholm area were invited to participate. The Head of each preschool received oral and written information about the study and was contacted again one week later. All invited preschools agreed to participate. In total 30 children, aged four years +/- 6 months, were included after their parents had given written informed consent. No child had any impairment that affected their participation in physical activity. The study was approved by the Regional Ethical Review Board in Stockholm County, Dnr 2009/217-31/2.

### Procedures

The calibration took place at each preschool in the morning hours between 8–11 am. While being barefoot, children’s weight was measured with an electronic scale (Tanita HD-316, Tanita Corp.; Tokyo, Japan) and height with a portable stadiometer. Body mass index (BMI) was calculated and children were classified as either normal weight or overweight [12]. Each child was equipped with two Actigraph GT3X+ accelerometers, one with a nylon wrist band on the non-dominant wrist [5, 11], determined by the preschool staff, and the other attached with an elastic band on the left hip. A maximum of six children at a time performed three indoor activities (sitting watching a cartoon, sitting drawing, dancing to music) and one free-play outdoor session. The activities chosen were considered representative of 4-year old children and were selected after discussions with staff at one preschool. Each indoor session was performed for 4–6 minutes and the outdoor session lasted for about 15 minutes. Each session was video recorded (Canon Inc., Tokyo, Japan). Subsequently, physical activity intensity levels for each child were coded according to CARS [8], every 5^th^ second by watching the videos. The scoring was performed by one researcher who had received several hours of training prior to the scoring session. In case of uncertainty the scoring researcher had the possibility to discuss together with another researcher with experience of using CARS. By video recording the computer clock on that same computer that was used for initializing the accelerometers in the beginning of each session, accelerometer data and CARS score could subsequently be synchronized.

### Accelerometry

The Actigraph GT3X+ accelerometer (Actigraph LLC, Pensacola, FL, USA) is a small (45x33x15 mm), lightweight (19 g) monitor. It is water resistant and can be worn on various body sites including the wrist, hip and ankle. Data is sampled in a pre-determined interval and summarized over a so-called epoch. In this study, accelerometer data was sampled at 30 Hz and summarized over 5-second epochs. A 5-second epoch has been recommended to be used in order to capture the short bursts of MVPA that characterize preschool children’s physical activity [3]. The Actigraph GT3X+ accelerometer is three-axial, meaning that accelerations are captured in three directions. The VM is a combined measure of the three axes, defined as $\sqrt{x^{2}+y^{2}+z^{2\;}}$. Whether the VA or the VM gives the most accurate measure of physical activity remains the subject of ongoing debate [13].

### CARS

CARS is an observational method that has been used in its original form, as well as in modified versions, in previous calibration studies and deemed appropriate for this study [5, 9, 14]. The method allows an observer to score the physical activity intensity for a child on a scale from 1–5. Level 1 represents only minor movements; Level 2 standing, moving arms; Level 3 walking slowly; Level 4 walking briskly; and Level 5 represents running. A detailed manual is available [8].

### Data analysis

A CARS score of 1 was considered sedentary, CARS scores 2 and 3 were considered light intensity and 4 and 5 a proxy for MVPA [5, 8, 15]. Receivers Operating Characteristic (ROC) curves were used to develop wrist and hip upper intensity threshold for sedentary and lower thresholds for MVPA, for the VA and for the VM. Using ROC curve analysis, accelerometer count thresholds are chosen based on sensitivity and specificity and it is a method commonly used in calibration studies [5, 16]. When using ROC curve analysis, a measure of the area under the curve (AUC), representing the classification accuracy is provided. An AUC of under 0.70 is considered as poor, 0.70–0.79 fair, 0.80–0.89 good, and at least 0.90 excellent [17]. A leave-one-out method was used to cross-validate the derived intensity thresholds. Data from all of the participants except one, which was “held out”, was used as the test dataset. The process was repeated until all participants had served as the test data set and the accuracy results were averaged. ROC analysis was conducted on n-1 participants, followed by determination of agreement between the numbers of 5-second epochs at each intensity by developed cut points. This was compared with the number of 5-second epochs in each CARS score of the left-out child. This was repeated throughout the total sample. Absolute agreement and quadratic weighting Kappa was calculated [18]. The previously developed MVPA intensity thresholds for children aged 3–5 by Pate et al. [19] and Sirard et al. [10] were compared against the output from our VA ROC curves for the hip in terms of sensitivity and specificity. Since a 15-second epoch length was used in these studies, these count thresholds were divided by three before being applied to the output from the ROC curves. The MVPA intensity threshold was then ≥140 counts/5 seconds and ≥271 counts/5 seconds for the Pate et al. and the Sirard et al. intensity thresholds, respectively.

## Results

Table 1 shows descriptive characteristics of the included children. Of the 30 children, 14 were boys. One fifth was considered overweight, but no child was obese.

**Table 1:** Child descriptive characteristics. n = 30.

	Mean (SD)	N (%)
Age (months)	49 (3.7)	
Sex (% boys)		14 (47)
Weight (kg)	17.6 (2.0)	
Height (cm)	104 (4)	
BMI (kg/m^2^)	16.3 (1.1)	
Weight status (% overweight)		6 (20)

Average observed time was 29 (SD 6) minutes per child. Mean accelerometer counts were lowest when children were watching a cartoon and highest when dancing to music (Table 2).

**Table 2:** Mean (SD) counts per 5 seconds for wrist and hip by activity (n = 30).

	Vertical axis		Vector magnitude	
*Activity*	*Wrist*	*Hip*	*Wrist*	*Hip*
Cartoon	35 (33)	3 (4)	91 (73)	14 (15)
Drawing	129 (55)	14 (14)	276 (85)	54 (32)
Dancing	634 (209)	220 (93)	1093 (330)	396 (148)
Outdoor	351 (176)	92 (57)	588 (267)	209 (86)

Pursuant to the protocol, mean accelerometer counts increased by CARS score (Table 3).

**Table 3:** Mean counts per 5 seconds for wrist and hip by CARS score. n = 30.

CARS score		*Wrist*	*Hip*
1	Vertical axis	170 (65)	43 (33)
	Vector magnitude	317 (105)	103 (60)
2	Vertical axis	303 (88)	77 (47)
	Vector magnitude	543 (128)	181 (81)
3	Vertical axis	508 (123)	176 (82)
	Vector magnitude	865 (191)	354 (128)
4	Vertical axis	934 (303)	329 (150)
	Vector magnitude	1559 (462)	505 (205)
5	Vertical axis	1184 (499)	577 (304)
	Vector magnitude	2129 (740)	774 (340)

Table 4 shows the developed intensity thresholds together with sensitivity, specificity and AUC.

**Table 4:** Sensitivity, specificity, area under the curve (AUC) and intensity threshold counts per 5 seconds for wrist and hip.

	Intensity level†	Sensitivity(%)	Specificity(%)	AUC(95% CI)	Intensity threshold(5 s)
*Wrist*					
Vertical axis	Sedentary	100	60	0.99 (0.97–1.0)	≤ 178
	Light				179–870
	MVPA	70	100	0.95 (0.9–1.0)	≥ 871
Vector magnitude	Sedentary	100	60	0.99 (0.97–1.0)	≤ 328
	Light				329–1392
	MVPA	70	100	0.96 (0.91–1.0)	≥ 1393
*Hip*					
Vertical axis	Sedentary	100	60	0.93 (0.86–1.0)	≤ 43
	Light				44–289
	MVPA	70	100	0.95 (0.86–1.0)	≥ 290
Vector magnitude	Sedentary	100	60	0.95 (0.89–1.0)	≤ 105
	Light				106–511
	MVPA	70	93	0.91 (0.83–0.99)	≥ 512

^†^Sedentary based on CARS score 1; Light based on the average of CARS scores 2 and 3; MVPA based on the average of CARS scores 4 and 5.

Sensitivity was 100% and 70%, and specificity was 60% and 93–100% for the sedentary and MVPA intensity thresholds, respectively. AUC ranged from 0.91–0.99.

The absolute agreement was 82% (VA and VM) for the wrist-worn accelerometer and 68% (VA) and 75% (VM) for the accelerometer worn on the hip. For the wrist-worn accelerometer, the quadratic weighted Kappa was almost perfect; 0.92 (95% CI 0.91–0.93) and 0.95 (95% CI 0.94–0.96) for the VA and the VM, respectively. The Kappa values for the hip-worn accelerometer were lower compared to the wrist-worn device; 0.76 (98% CI 0.74–0.77) and 0.86 (95% CI 0.85–0.87) for the VA and VM, respectively.

When applying the Pate et al. intensity thresholds to our VA ROC curve for the hip, sensitivity and specificity for the MVPA intensity threshold were 97% and 67%, respectively. For the Sirard et al. threshold, sensitivity and specificity were 73% and 97%.

## Discussion

This is the first study to develop intensity thresholds for both the VA and the VM for a wrist-worn Actigraph accelerometer in preschool children. A previous study has been performed in two-year-olds, using an almost identical protocol as in this study [20], as well as in 8–12 years olds [11]. Compared to those, the intensity thresholds developed in this study were higher. This indicates that age-specific thresholds are needed. During the infant, toddler and preschool years, motor skills are developing rapidly. The gait becomes increasingly stable which enables the child to move around more effectively than at earlier ages. Arm movements decrease gradually as the child ages, explaining the higher count thresholds in comparison with the study on 8–12 year olds [11]. In comparison with these studies, we found higher measures of AUC, sensitivity and specificity for the developed intensity thresholds.

Two previous studies have developed intensity thresholds for the hip-worn uniaxial Actigraph accelerometer for four-year-old children [10, 19]. In the study by Pate et al. oxygen consumption was used as the criterion measure and children performed structured activities during the calibration session [19]. In the study by Sirard et al. CARS was used as the reference method, as in the present study. Using the same definition of MVPA (CARS score of at least 4), the developed count thresholds were similar to those derived in this study [10], confirming the validity of our hip MVPA intensity threshold. When applying the intensity thresholds by Pate et al. and Sirard et al. to the output from the ROC curves from this study, the sensitivity and specificity were almost the same in our study as for the MVPA threshold developed by Sirard et al. In order to avoid overestimation of the time spent in MVPA, we chose an intensity threshold with high specificity, at the expense of a lower sensitivity for that threshold. For the threshold developed by Pate et al., however, the sensitivity was high but the specificity relatively low, which will lead to an overestimation of time spent in MVPA. This indicates that the choice of criterion measure and definition of moderate intensity have great impact on the intensity thresholds derived.

The definition of moderate intensity is under debate and has been defined differently across studies [11, 21]. Like in previous studies [9, 10, 14] CARS scores 4 and 5 were categorized as a proxy for MVPA. This is based on a validation study, in which CARS was validated against oxygen consumption in 5-6-year-olds. In that study, CARS score 3 and 4 was found to correspond to 2.69 and 3.45 metabolic equivalents, respectively [8]. CARS has also been found to accurately classify the level of energy expenditure and to correlate with Caltrac motion sensors in children aged 2–6 [15]. Nevertheless, estimating what represents moderate intensity in preschoolers is problematic. It is known that the energy cost for physical activity per kilo body weight tends to decrease with advancing age [22]. Since the ability to absorb oxygen, muscle strength and motor skills develop as the child mature, it is likely that the metabolic equivalent for a certain activity differs between a four- and a six-year-old. This area of research needs further exploration.

Since a three axial accelerometer was used in the present study, intensity thresholds for the VM were also developed. These were found to have higher validity than those for the VA (Kappa value of 0.95 vs 0.92 for the wrist and 0.86 vs 0.76 for the hip). This is in accordance with a previous study, which found that activity counts derived from the VM yielded a more accurate estimation of energy expenditure than those derived from the VA, in youths, adults and older adults [23]. In the present study, the VM also had higher validity for the wrist intensity thresholds, as was found in a previous study on two-year-olds [5]. Thus, placing the Actigraph on the wrist and using the VM may be recommended for assessing time in different physical activity intensities in this population.

The accelerometer output differs according to the body site to which it is attached. A wrist-worn accelerometer captures movements performed by the arms, unlike a hip-mounted monitor. In accordance with results from previous studies, we found that accelerometer data collected from the wrist yielded higher accelerometer output compared with the hip-worn monitor, while measuring the same individual concurrently [6, 7]. Previous studies on adults have demonstrated that hip-mounted monitors have better accuracy in predicting physical activity energy expenditure than wrist-mounted monitors [24–26]. Wearing the accelerometer on the wrist can be problematic if the subject is walking with the hands constrained, for example when carrying a load. The accelerometer output will probably then be much different from walking with the hands swinging freely. However, when observing children during free play children tend to engage in activities like carrying heavy objects infrequently, making this likely to represent a minor problem among the preschool population. A wrist placement has been shown to accurately estimate energy expenditure [27] and, as in the present study, to estimate time spent in different intensities [5, 11] in children. Since a wrist placement can increase compliance [6], these results are promising.

### Strengths and limitations

Strengths of the present study are the use of a commonly used reference method to categorize physical activity into intensity levels, including both structured activities and free play. Data were summarized over a short epoch length of 5 seconds, which has been recommended for young children [3]. The study design enables comparison of the VA and the VM, as well as wrist and hip placement of the accelerometer, helping researchers and practitioners chose the optimal monitor placement site and data handling for assessing time in physical activity intensities for this population.

Some limitations need to be discussed. First, one fifth of the included children were considered overweight, but none were obese. The output does not seem to differ between adults with different BMI [28] but it is possible that our results would have been different with a more heterogeneous sample. Secondly, accelerometer and CARS data were matched on a 5-second level because children in this age group change their activity level frequently. This is a very narrow time span making it possible that data were mismatched. Hence, by video recording the computer clock on that same computer that was used for initializing the accelerometers in the beginning of each session, we ensure that the correct 5 seconds were matched. Third, when using ROC curve analysis, the intensity thresholds were chosen based on sensitivity and specificity. A high sensitivity was chosen at the expense of low specificity, and vice versa. Since we did not want to underestimate sedentary time, we chose to prioritize a high sensitivity for that threshold. In the same way, we did not want to overestimate time in MVPA, which is why high specificity was prioritized for the MVPA thresholds. A high specificity for the MVPA threshold is important in order to correctly evaluate if a child is reaching recommendations for physical activity. This approach can, however, be questionable. Regarding external validity, only healthy children without impairments were included, which is why these results should not be generalized to populations of children with disabilities.

## Conclusion

The derived thresholds were found to be valid for categorizing physical activity intensity in four-year-old children. For both wrist and hip placement the VM had higher validity than the VA. In comparison with placing the accelerometer on the hip, the wrist-mounted monitor performed better in accurately assessing time spent being sedentary, in light activity and in MVPA. Based on the findings of this study, the VM based on wrist-worn accelerometers for measuring physical activity in 4-year-old children is recommended to use.
